# Targeting Grancalcin Accelerates Wound Healing by Improving Angiogenesis in Diabetes

**DOI:** 10.1002/advs.202305856

**Published:** 2024-02-02

**Authors:** Peng Xiang, Meng Jiang, Xin Chen, Linyun Chen, Yalun Cheng, Xianghang Luo, Haiyan Zhou, Yongjun Zheng

**Affiliations:** ^1^ Department of Endocrinology Endocrinology Research Center Xiangya Hospital of Central South University Changsha Hunan 410008 China; ^2^ National Clinical Research Center for Geriatric Disorders Xiangya Hospital Changsha Hunan 410008 China; ^3^ Department of Burn Surgery the First Affiliated Hospital of Naval Medical University Shanghai 200433 China

**Keywords:** angiogenesis, bone marrow‐derived cell, diabetic wound, grancalcin‐neutralizing antibody, hydrogel

## Abstract

Chronic diabetic wounds are a serious complication of diabetes and often result in limb amputations and confer high mortality rates. The proinflammatory secretome in the wound perpetuates defective neovascularization and contributes to dysregulated tissue repair. This study aims to design a gelatin methacrylamide (GelMA) hydrogel to sustained the release of grancalcin‐neutralizing antibody (GCA‐NAb) and evaluate it as a potential scaffold to promote diabetic wound healing. Results show that the expression of grancalcin(GCA), a protein secreted by bone marrow‐derived immune cells, is elevated in the wound sites of individuals and animals with diabetic ulcers. Genetic inhibition of grancalcin expression accelerates vascularization and healing in an animal model. Mechanistic studies show that grancalcin binds to transient receptor potential melastatin 8(TRPM8) and partially inactivates its downstream signaling pathways, thereby impairing angiogenesis in vitro and ex vivo. Systemic or topical administration of a GCA‐NAb accelerate wound repair in mice with diabetes. The data suggest that GCA is a potential therapeutic target for the treatment of diabetic ulcers.

## Graphical abstract

In diabetic ulcer‐afflicted mice, bone marrow‐derived GCA^+^ immune cells release substantial amounts of grancalcin(GCA), hindering diabetic wound healing through angiogenesis inhibition. To counter this, a hydrogel embedded with GCA‐neutralizing antibodies(GelMA‐GCA‐NAb) specifically for treating diabetic wounds is developed. Using GelMA‐GCA‐NAb notably expedites wound closure, augments angiogenesis, and mitigates local inflammatory responses.

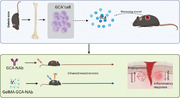

## 1 Introduction

Diabetes is a chronic metabolic disorder that has affected ≈537 million people worldwide.^[^ ^1^ ^]^ Non‐healing wounds, such as diabetic foot ulcer (DFU), are common in patients with diabetes and are usually accompanied by vascular complications. ≈25% of all patients with diabetes develop DFU during their lifetime.^[^ ^2^ ^]^ DFUs significantly impair the quality of life, increase morbidity and mortality and impose a substantial economic burden on healthcare resources. The bone marrow‐derived cell (BMDC) induces paracrine release of cytokines, exosomes, and growth factors to promote tissue regeneration and wound repair.^[^ ^3^ ^]^ The therapeutic effects of BMDCs in diabetes‐related vascular complications, including DFUs,^[^ ^4^ ^]^ diabetic nephropathy,^[^ ^5^ ^]^ and limb ischemia,^[^ ^6^ ^]^ have been reported. Although most of these studies focused on the beneficial BMDC‐induced paracrine factors, several studies have indicated that BMDCs from animals and individuals with diabetes evince a major beneficial‐to‐harmful alteration in their secretome components.^[^ ^5^ ^]^ Changes in the secretory profile of BMDCs impair their capacity to ameliorate diabetes‐related complications.^[^ ^5^, ^7^, ^8^ ^]^

Angiogenesis is a rate‐limiting step in wound healing.^[^ ^9^ ^]^ Neovascularization abnormalities are usually present in patients with diabetes.^[^ ^10^ ^]^ Bone marrow‐derived immune cells accumulate in chronic unhealing wounds and secrete proinflammatory cytokines, which stall healing in the inflammatory phase and make it difficult to proceed to the repairing phase.^[^ ^11^, ^12^, ^13^ ^]^ Inhibition of the proinflammatory secretome accelerates neovascularization and wound healing.^[^ ^14^, ^15^, ^16^ ^]^ Thus, identifying the key proinflammatory secretome responsible for neovascularization may offer an effective strategy for the treatment of diabetic wounds. In a previous study, we reported that GCA, a secreted protein from proinflammatory and senescent subtypes of myeloid cells, induces skeletal aging by suppressing bone turnover and promoting marrow fat accumulation.^[^ ^17^ ^]^ Given the critical role of the proinflammatory secretome in angiogenesis and tissue regeneration, we hypothesized that the myeloid cell‐derived secretory protein‐GCA may be involved in the regulation of wound healing in diabetes.

In this study, we aimed to determine whether the genetic deletion of GCA in myeloid cells improves angiogenesis and wound healing in diabetes. We systematically administered an intravenous GCA‐NAb to improve wound repair in diabetic mice. To further explore the efficacy of local antibody administration, GCA‐NAb was coated with a hydrogel and administered to excisional wounds. Consistent with the systemic application, we checked if topical application of GCA‐NAb‐loaded hydrogels accelerates wound closure in diabetes. Our findings will lay a scientific foundation for future studies and suggest a potential therapeutic target for the treatment of diabetic ulcers.

## 2 Results

### 2.1 GCA Deficiency Accelerates Wound Healing in Diabetes

**Figure 1:**
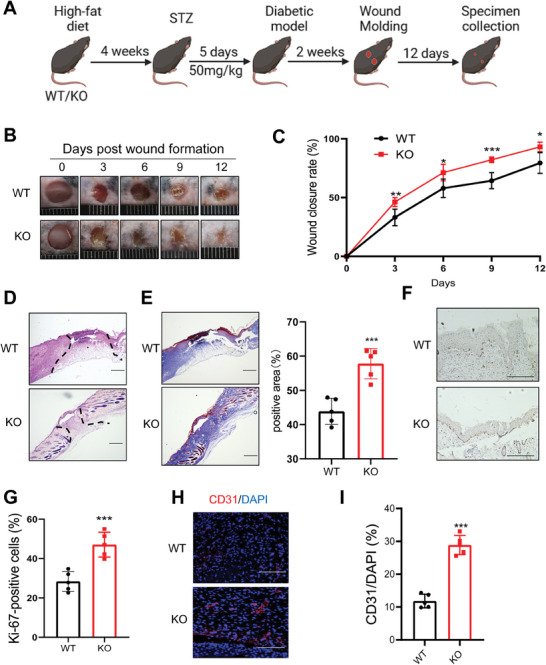
GCA deficiency accelerates wound healing in diabetes. A) The process of the diabetic wound molding model in wild‐type (WT) and GCA knockout (KO) mice (n = 5). Representative wound photographs B) and wound‐healing rates C) at days 0, 3, 6, 9, and 12. D) Representative hematoxylin–eosin‐stained wound sections on Day 12 (scale bars, 200 µm). E) Representative image of Masson's trichrome staining of the wound sections on Day 12 (scale bars, 200 µm). F,G) Immunohistochemical staining and quantitative analysis of Ki67 expression in wound sections on Day 12 (scale bars, 100 µm). Immunofluorescence staining H) and quantitative analysis of CD31 I) expression at the wound site on Day 12 (scale bars, 100 µm). Data are shown as the mean ± SD. ^*^ *p* < 0.05; ^**^ *p* < 0.01; ^***^ *p* < 0.001; ^****^ *p* < 0.0001.

We discovered that GCA‐knockout(KO) mice showed faster postoperative wound recovery; therefore, we further explored whether GCA plays an important role in chronic wound healing (**Figure** **1**A). Compared to WT mice, KO mice had faster wound closure, suggesting that GCA deficiency accelerated the healing process (Figure 1B,C). Histological comparison of the wounds further confirmed the accelerated wound closure in KO mice (Figure 1D). Trichrome staining of the tissue harvested on Day 12 after wound infliction showed increased collagen content in the granulation tissue of KO mice (Figure 1E). Staining for Ki67 indicated considerably more proliferating cells in the wound areas of KO mice than in WT mice (Figure 1F,G). Similarly, the intensity of CD31‐positive immunostaining was elevated in wound tissues of KO mice, suggesting that GCA deficiency enhanced angiogenesis during the wound‐repair process in diabetes (Figure 1H,I). Collectively, these data suggest that GCA deficiency accelerates wound repair and regeneration by regulating collagen deposition and angiogenesis.

### 2.2 GCA Levels were Increased in the Wound Tissues of Patients and Mice with Diabetes

**Figure 2:**
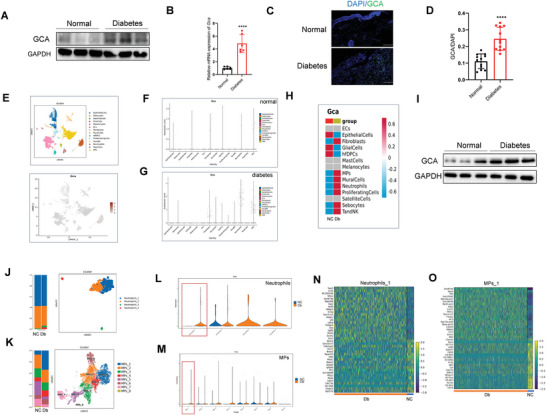
Enrichment of GCA in myeloid cells of diabetic ulcers. A) Western blot analysis of GCA in normal skin tissue and tissue from diabetic ulcer (n = 3); GAPDH was used as a loading control B) The mRNA levels of GCA were analyzed by qRT‐PCR in human skin tissue from diabetic ulcer (n = 6). Immunofluorescence staining C) and quantitative analysis D) of GCA in human skin tissue(n = 12; scale bars, 200 µm). E) Bioinformatic analysis of scRNA‐seq of wound‐derived skin tissues from normal and diabetic mice. Violin plots of log‐transformed expression of GCA‐related genes in the cell populations of normal F) and diabetic G) mice. H) Heatmap of GCA expression in the wound‐derived skin cells of normal and diabetic mice. I) Western blotting was performed to detect GCA expression in bone marrow‐derived cells (BMC) from normal and diabetic mice; GAPDH was used as a loading control (n = 3). T‐distributed stochastic neighbor embedding (tSNE) plot showing the clustering of neutrophils J) and MPs K) based on GCA expression. Violin plots of GCA expression in clusters of neutrophils L) and MPs M). Highly expressed differentially expressed genes in neutrophil_1 N) and MPs_1 O) clusters in normal and diabetic mice. Data are shown as the mean ± SD. ^****^ *p* < 0.0001.

Compared to that in normal skin samples, we identified a pronounced increase in GCA expression in the skin of patients with diabetic ulcer (**Figure** **2**A–D). Accordingly, GCA expression at the wound sites (Figure S1A–C, Supporting Information) in diabetic mice increased as compared with those in normal wild‐type mice, thereby supporting a potentially significant role of GCA in diabetic wound healing.

### 2.3 Enrichment of GCA in Myeloid Cells of Diabetic Ulcers

Bioinformatics analysis using scRNA‐seq suggested that GCA was enriched in myeloid cells (Figure S1D,E, Supporting Information). To identify the primary sources of GCA in skin tissues from diabetic wounds, we conducted single‐cell sequencing of skin tissues from immune cells, particularly macrophages and monocytes (MPS) and neutrophils (Figure 2E). We observed significantly higher GCA expression in the neutrophils and MPS of diabetic mice of GCA origin than in normal mice (Figure 2F–H). Subsequent tests revealed a notable increase in GCA expression in bone marrow‐derived cells from diabetic mice as compared to those from normal mice (Figure 2I). This finding suggests a potential association between immune cell‐derived GCA and the pathophysiology of diabetic ulcers.

To gain a deeper understanding of GCA^+^ MPs and neutrophils, we performed single‐cell transcriptional profiling using the scRNA‐seq database to identify distinct cell populations. This analysis revealed the presence of multiple transcriptionally distinct clusters, allowing further characterization of these cell populations. Among these, clusters MPS_1 and neutrophil _1 exhibited the highest numbers (Figure 2J,K). Notably, GCA expression was significantly higher in the MPS_1 and Neutrophil_1 clusters in diabetic mice than that in normal mice (Figure 2L,M). We analyzed the transcriptional profiles of the MPS_1 and Neutrophil_1 clusters to understand their molecular differences and potential implications for diabetic wound healing. We found that diabetic mice overexpressing GCA^+^ cells exhibited significant proinflammatory phenotypes (Figure 2N,O). These results indicate that GCA in diabetic ulcers primarily originates from myeloid cells and that GCA plays a crucial role in wound healing.

### 2.4 Myeloid‐Specific GCA Deficiency Enhances Angiogenesis in Wound Sites

**Figure 3:**
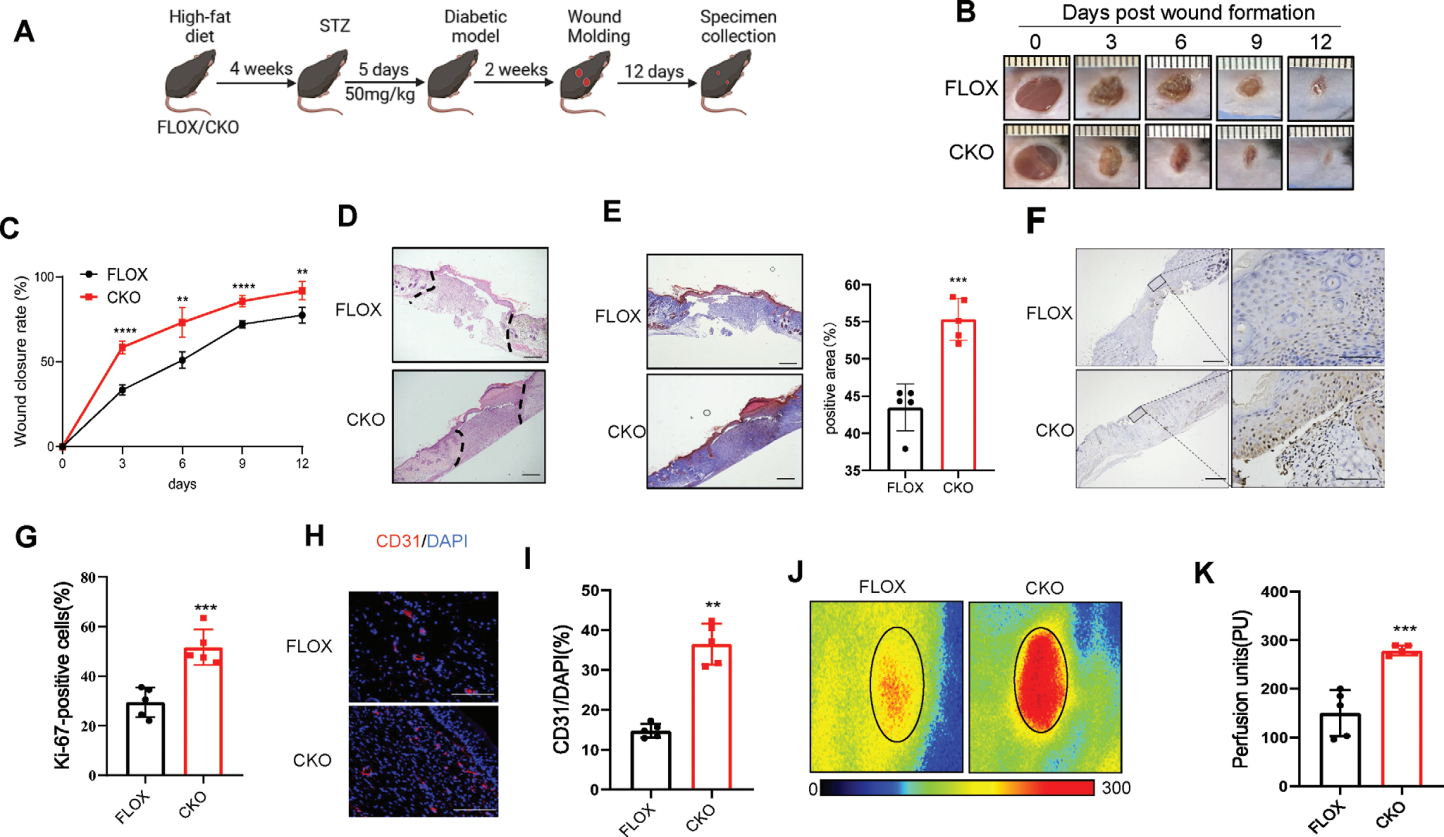
Myeloid‐specific GCA knockout accelerates wound healing in diabetic mice. A) Molding of diabetic wounds in FLOX and CKO mice(n = 5). Representative wound photographs B) and wound‐healing rates C) at days 0, 3, 6, 9, and 12. D) Representative hematoxylin–eosin‐stained wound sections on Day 12 (scale bars, 200 µm). E) Representative image of Masson's trichrome staining of the wound sections on Day 12 (scale bars, 200 µm). Immunohistochemical staining E) and quantitative analysis F) of Ki67 expression in the wound sections on Day 12 (scale bars, 200 and 50 µm). Immunofluorescence staining H) and quantitative analysis I) of CD31 in wounds on Day 12 (scale bars, 100 µm). Representative images of the dorsal skin were captured using a laser Doppler imager for each group J). Quantitative analysis of perfusion K) of the back skin in mice is shown 7 days post‐injury (the solid circle indicates the wound bed in each group of mice). Data are shown as the mean ± SD. ^**^ *p* < 0.01; ^***^ *p* < 0.001; ^****^ *p* < 0.0001.

GCA is a paracrine‐acting protein that is secreted mainly from bone marrow‐derived cells; however, myeloid cells are not the only GCA‐expressing cell type (Figure 2F–H). We used myeloid‐specific GCA‐knock‐out (CKO) mice to address the importance of bone marrow cell‐derived GCA in wound healing in diabetes (**Figure** **3**A). GCA expression in wounds was significantly lower in CKO mice than in WT mice (Figure S6C, Supporting Information). As expected, myeloid‐specific GCA deficiency accelerated wound closure in the diabetic animals (Figure 3B,C).

Histological analysis using hematoxylin and eosin (H&E)staining (Figure 3D) showed that myeloid GCA deficiency had comparable beneficial effects on dermal and epidermal regeneration. Masson's trichrome staining indicated that GCA deficiency induced obvious changes in collagen deposition at wound sites (Figure 3E). Immunohistochemical staining for Ki67 revealed that cell proliferation in CKO mice was remarkably enhanced as compared to WT mice (Figure 3F,G). These findings suggest that the ablation of GCA in myeloid cells is sufficient to accelerate the healing of diabetic wounds.

Considering the important role of angiogenesis in wound healing, we investigated whether GCA deficiency influenced angiogenic activities in the wound area. The levels of angiogenesis‐related genes were significantly elevated in the wound area of the CKO mice (Figure S2A,B, Supporting Information). Dermal microvessels were assayed for the endothelial marker CD31, which showed that myeloid GCA deficiency significantly increased microvessel density at the wound sites in diabetic mice (Figure 3H,I). Skin‐perfusion pressure tests indicated that myeloid GCA deficiency significantly enhanced the skin blood‐flow rate 6 days post‐injury in animals with diabetes (Figure 3J,K). Wound inflammation significantly improved in CKO mice (Figure S2C, Supporting Information). Taken together, these results indicated that myeloid GCA deficiency adequately improved angiogenesis and wound repair in diabetic mice.

### 2.5 GCA Impairs Tube Formation and Migration in Endothelial Cells

**Figure 4:**
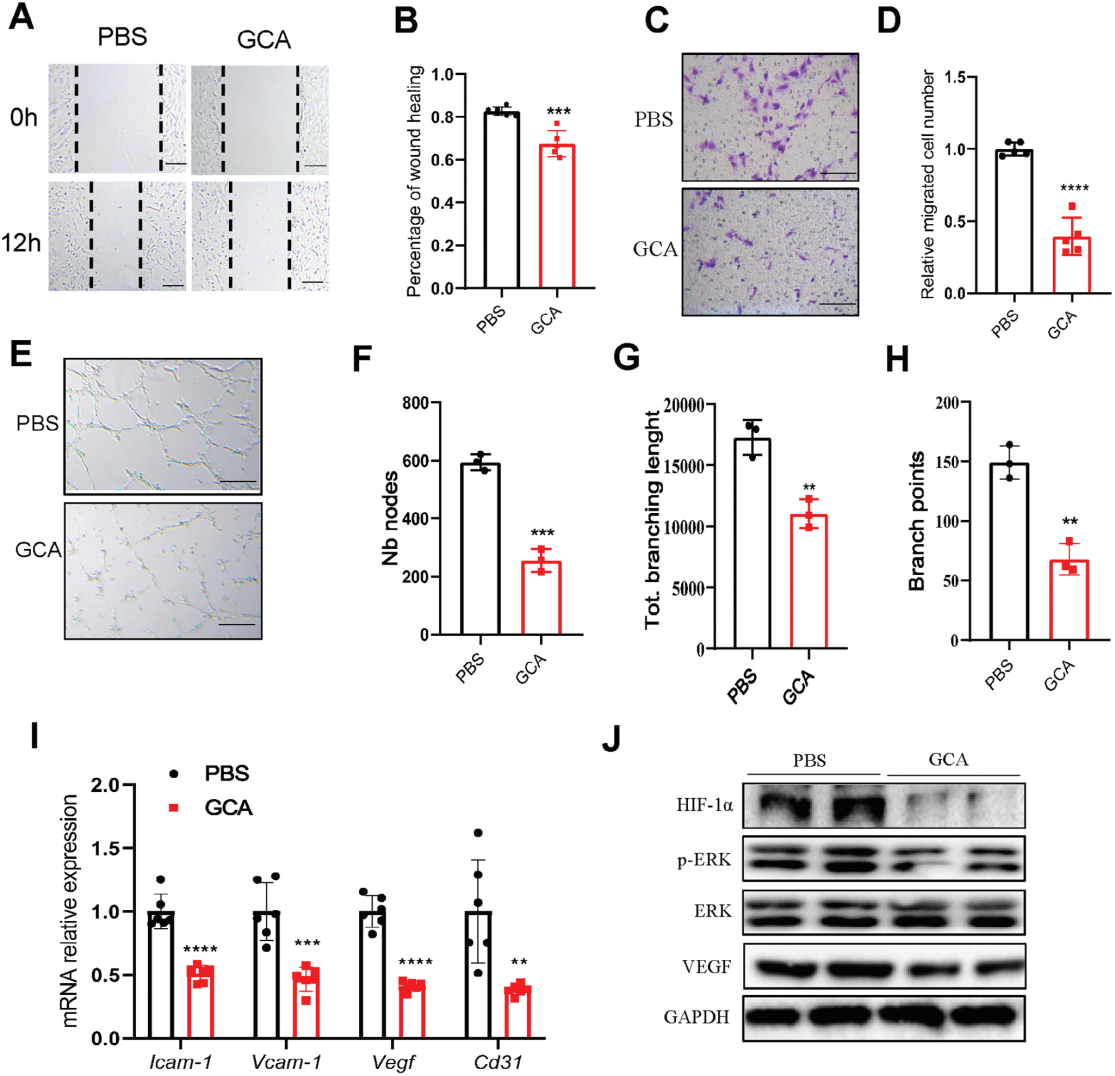
GCA impair tube formation and migration in endothelial cells. GCA‐induced functional changes in HUVEC, including migration, tube formation, and the expression of various angiogenic factors. Live cell images A) were captured 12 h after the wound‐healing assay (scale bars, 50 µm), and the wound recovery rate B)was quantified using ImageJ (n = 3). Representative images C) of invasive cells in the lower chamber that were stained with crystal violet and obtained to evaluate the migration ability of HUVECs (n = 3) in the PBS and GCA groups (scale bars, 100 µm). Quantification of migration ability is shown in the histogram D). Representative images E) of the tube‐formation assays were taken after 6 h, with or without GCA treatment (scale bars, 100 µm); Nb nodes F), total branching length F), and number of branch points H) were measured and analyzed using ImageJ (n = 3). (I) Quantification of angiogenesis‐related gene expression in HUVEC after 24 h GCA treatment. J) Protein expression levels of HIF‐1α, p‐Erk1/2, and VEGF were analyzed by Western blot in HUVECs cultured under GCA or PBS for 48 h. GAPDH as a loading control. Data are shown as the mean ± SD. ^**^ *p* < 0.01; ^***^ *p* < 0.001; ^****^ *p* < 0.0001.

To further verify whether myeloid‐derived GCA is a paracrine factor that regulates endothelial angiogenesis, we conducted in vitro experiments using HUVECs. Based on our previous study, we used 100 nM rGCA as the challenge concentration. The scratch wound‐healing test showed that rGCA treatment significantly impaired HUVEC migration (**Figure** **4**A,B). The negative effect of rGCA on endothelial cell migration was further confirmed using a Transwell migration assay (Figure 4C,D). Consistently, rGCA treatment decreased the ability of HUVECs to form vessel‐like structures, as shown by the tube‐formation assay (Figure 4E–H). Additionally, the mRNA levels of angiogenesis‐related genes (Icam‐1, Vcam‐1, Vegf, and Cd31) significantly decreased (Figure 4I) in GCA‐treated endothelial cells. Thus, rGCA treatment impairs the angiogenic activity of endothelial cells.

### 2.6 TRPM8 is a GCA Receptor in Endothelial Cells

**Figure 5:**
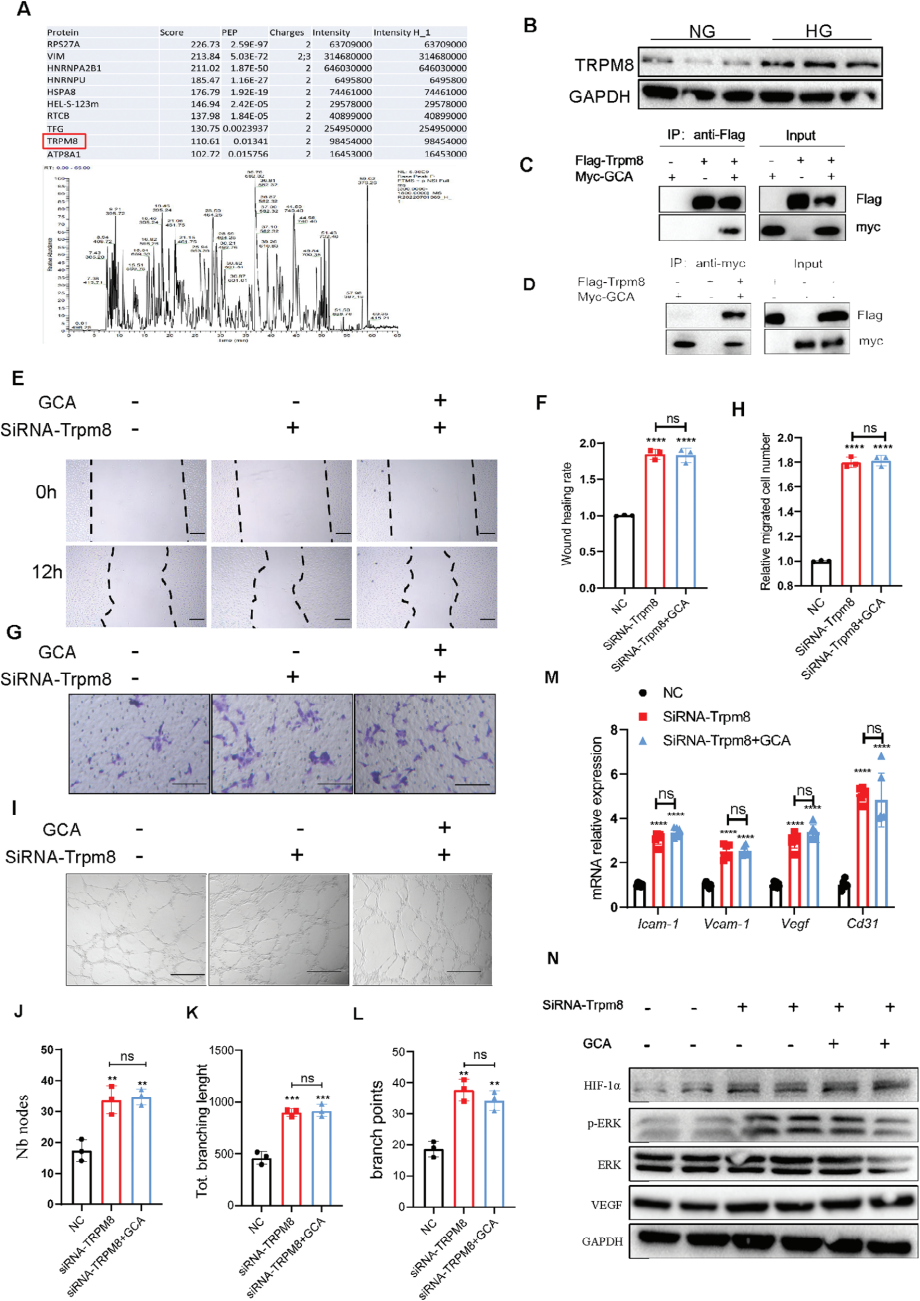
TRPM8 is a receptor of GCA in endothelial cells. A) TRPM8 protein binding to GCA was identified by LC‐MS/MS analysis. B) TRPM8 expression in HUVECs was determined by Western blotting after incubation with normal or high glucose. Immunoprecipitation (IP) analysis of Flag‐TRPM8 C) and Myc‐GCA D) binding. GCA and siRNA‐Trpm8 treatments induced functional changes in HUVECs (n = 3), including migration, tube formation, and expression of various angiogenic factors. Live cell images E) were captured 12 h after the wound‐healing assay (scale bars, 100 µm), and the wound‐recovery rate F) was quantified using ImageJ. Representative images of invasive cells G) in the lower chamber stained with crystal violet were obtained to evaluate the migratory ability of HUVECs(scale bars, 100 µm); and quantitative analysis H)of migration ability. Representative images of HUVEC tube formation I)(scale bars, 100 µm). Nb nodes J), total branching length K), and number of branch points L) were measured and analyzed using ImageJ. M) mRNA expression of vasculogenesis‐related genes. N) Protein expression levels of HIF‐1α, p‐Erk1/2, and VEGF were analyzed by Western blot, with GAPDH as a loading control. Data are presented as mean ± SEM. NS, not significant;^**^ *p* < 0.01; ^***^ *p* < 0.001; ^****^ *p* < 0.0001.

To determine the potential GCA receptors in endothelial cells, we used mass spectrometry to analyze proteins isolated from endothelial cells incubated with His‐GCA. We used the top ten proteins from the liquid chromatography‐tandem mass spectrometry (LC‐MS/MS) analysis for further studies (**Figure** **5**A). TRPM8 regulates the angiogenic activity of endothelial cells and wound healing.^[^ ^18^, ^19^, ^20^ ^]^ High glucose levels stimulated TRPM8 expression in endothelial cells (Figure 5B). Therefore, TRPM8 was selected for further analysis.

To verify the interaction between GCA andTRPM8, the plasmids (Myc‐GCA and Flag‐TRPM8) were transfected into human embryonic kidney (HEK) 293T cells, and the cell lysate was collected for immunoprecipitation using an anti‐Myc antibody and western blotting using an anti‐Flag antibody. A strong band of Flag was observed in Myc immunoprecipitants but not in IgG controls (Figure 5C). We conducted an immunoprecipitation assay using an anti‐FLAG antibody and a western blot assay using an anti‐Myc antibody. We observed a strong band of Myc staining in FLAG immunoprecipitants (Figure 5D).

To prove that TRPM8 mediates the rGCA effects on the angiogenesis of endothelial cells, the endothelial cells were transfected with siRNA TRPM8 or siRNA control, and the angiogenic activity of endothelial cells was detected in the presence of GCA. TRPM8‐interference efficiency was measured with Western blotting (Figure S6A, Supporting Information). These results suggest that the negative effects of GCA on angiogenesis were abolished in the siRNA‐TRPM8 group (Figure 5E–L). Consistently, siRNA‐TRPM8 reversed the GCA‐induced downregulation of angiogenesis‐related genes. Thus, TRPM8 may be a functional receptor for GCA and may play a critical role in GCA‐based suppression of angiogenesis.

### 2.7 GCA Inhibits the ERK/HIF‐1α/VEGF Signaling Pathway via TRPM8

Previous studies indicated that impaired function of the HIF‐1α/VEGF pathway is a central pathogenic mechanism for compromised angiogenesis and delayed wound healing in diabetes.^[^ ^21^, ^22^, ^23^, ^24^, ^25^, ^26^, ^27^ ^]^ Additionally, ERK activity is the key regulator of the HIF‐1/VEGF pathway in a various of disease condition. ERK/HIF‐1α/ VEGF pathway was reported involve in angiogenesis in diabetes and myocardial ischemia. Zhang et al reported that 20(S)‐Protopanaxadiol improve wound healing via ERK/HIF‐1α/ VEGF pathway in diabetic mice.^[^ ^26^ ^]^

Next, we speculated that GCA‐TRPM8 regulates angiogenesis via the ERK/HIF‐1α/VEGF pathway in endothelial cells. Myeloid‐specific GCA deficiency upregulated the ERK/HIF‐1/VEGF pathway in the wound tissues of diabetic mice (Figure S2A, Supporting Information). Moreover, GCA‐NAb treatment stimulated the same signaling pathway in the wound sites of diabetic animals (Figures S5A and S9E, Supporting Information). These results were further verified using endothelial cells. Consistent with the in vivo results, compared to PBS‐treated cells, we found a significant decrease in VEGF‐A expression in GCA‐treated cells (Figure 4I,J). GCA treatment significantly decreased the expression of phosphorylated ERK1/2 and HIF‐1α in endothelial cells (Figure 4J). The effects of rGCA on the ERK/HIF‐1α/VEGF pathway were all abolished in the siRNA‐TRPM8 group (Figure 5N). These data suggest that GCA interacts with TRPM8 and regulates the ERK/HIF‐1/VEGF pathway in endothelial cells.

### 2.8 GCA‐neutralizing Antibody Ameliorates Angiogenic Activity and Accelerates Wound Healing in Diabetes

GCA‐NAb was developed as previously described.^[^ ^17^ ^]^ GCA derived from BMDC has the potential to impede endothelial cell migration (Figure S3A,B, Supporting Information) and tube formation (Figure S3D–F, Supporting Information). However, GCA‐NAb demonstrated therapeutic potential by counteracting these effects. In vivo experiments were conducted to determine the effects of GCA‐NAb on angiogenesis (Figure S4A,B, Supporting Information).

**Figure 6:**
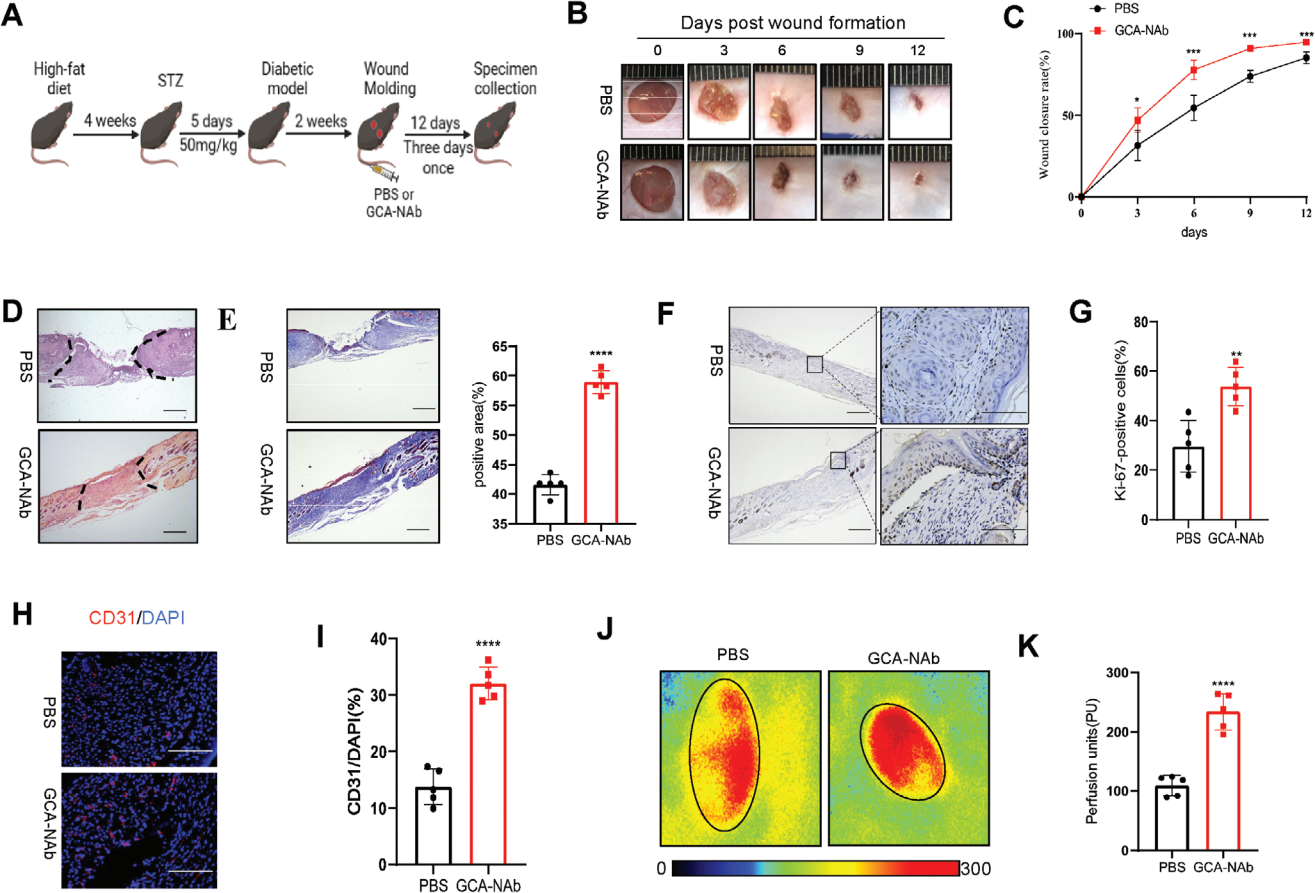
GCA‐NAb accelerates wound healing in diabetic mice. A) Schematic representation of the wound‐healing model for evaluating the therapeutic potential of GCA‐NAb. Representative wound photographs B) and wound‐healing rates C) at days 0, 3, 6, 9, and 12(n = 5). D) Representative hematoxylin–eosin‐stained wound sections on Day 12 (scale bars, 200 µm). E) Representative image of Masson's trichrome staining of the wound sections on Day 12 (scale bars, 200 µm). Immunohistochemical staining F) and quantitative analysis G) of Ki67 expression in the wound sections on Day 12 (scale bars, 200 and 50 µm). Immunofluorescence staining H) and quantitative analysis I) of CD31 in wounds on Day 12 (scale bars,100 µm). Representative images of the dorsal skin were captured using a laser Doppler imager for each group J), and quantitative analysis of perfusion (right) of the dorsal skin of mice is shown & days post‐injury (solid circle indicates the wound bed in each group of mice, n = 5). Data are shown as the mean ± SD. ^*^ *p* < 0.05; ^**^ *p* < 0.01; ^***^ *p* < 0.001; ^****^ *p* < 0.0001.

Diabetic mice were treated with GCA‐NAb (1 mg k^−1^g) via intravenous tail injection on alternate days for 2 weeks (**Figure** **6**A). Mice treated with GCA‐NAb showed a higher wound closure rate than vehicle‐treated controls (Figure 6B,C). On H&E staining (Figure 6D), mice treated with GCA‐NAb showed a much longer newly formed epidermis and dermis than mice treated with the vehicle. Masson's trichrome staining showed that GCA‐NAb treatment increased collagen deposition at wound sites (Figure 6E). Ki67 staining revealed the number of proliferating cells in the wound tissue of GCA‐NAb‐treated mice compared to that in vehicle‐treated mice (Figure 6F,G). These findings suggest that GCA neutralization accelerates the healing of diabetic wounds. The expression of angiogenesis‐related genes was significantly upregulated in the wound tissues of GCA‐NAb‐treated mice (Figure S5B, Supporting Information). CD31 immunostaining showed that GCA‐Nab treatment significantly increased vessel density at wound sites in diabetic mice (Figure 6H,I). Skin perfusion‐pressure assays suggested that GCA‐NAb treatment significantly enhanced the skin blood flow rate at 6 days post‐injury in diabetic mice (Figure 6J,K). Moreover, GCA‐nab significantly improved the inflammatory response in wounds (Figure S5C, Supporting Information). Taken together, these results indicated that GCA‐NAb treatment increased wound healing and angiogenesis in diabetic mice.

### 2.9 Topical Administration of GCA‐NAb‐loaded Hydrogel Enhances Wound Healing in Diabetic Mice

**Figure 7:**
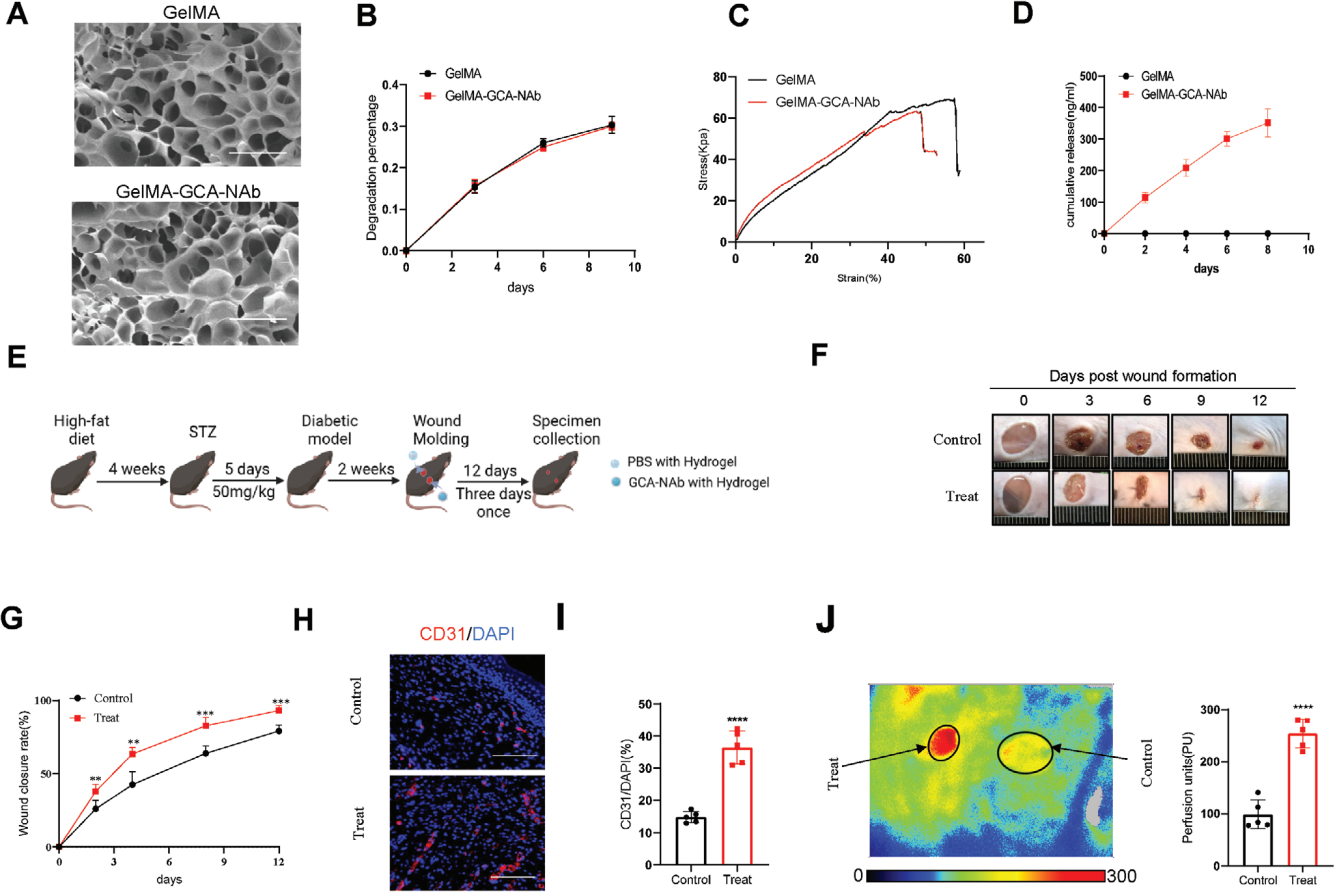
GCA‐neutralizing antibody‐embedded hydrogels enhance wound healing in diabetic mice. A)SEM images of GelMA and GelMA‐GCA‐NAb hydrogel(scale bars, 50 µm). B) Degradation percentage of GelMA and GelMA‐GCA‐NAb hydrogel (n  = 3). C)Stress of GelMA and GelMA‐GCA‐NAb hydrogel (n  = 3). D) Cumulative amount of protein released by GelMA‐GCA‐NAb(n = 3). E) Schematic depiction of the wound‐healing model for the evaluation of the therapeutic potential of GelMA‐GCA‐Nab (n = 5). Representative wound photographs F) and wound healing rates G) at days 0, 3, 6, 9, and 12.Immunofluorescence staining H) and quantitative analysis I) of CD31 in wounds on Day 12 (scale bars, 100 µm). Representative images of the dorsal skin were captured using a laser Doppler imager for each group.,and quantitative analysis of perfusion of the dorsal skin in miceJ) is shown 7 days post‐injury (the solid circle indicates the wound bed in each group of mice). Data are shown as the mean ± SD. ^**^ *p* < 0.01; ^***^ *p* < 0.001; ^****^ *p* < 0.0001.

To mitigate potential unknown systemic side effects of intravenous GCA‐NAb infusion while avoiding injection pain and infection, we developed a GCA‐NAb‐wrapped hydrogel for topical administration(Figure S7A–C, Supporting Information), aiming to enhance diabetic wound healing while considering clinical application. Different concentrations of GelMA‐GCA‐NAb hydrogels were designed, with 0.2 mg ml^−1^ GCA‐NAb concentration demonstrating the most effective therapeutic outcome(Figure S7D,E, Supporting Information).We analyzed multiple characteristics of GelMA‐GCA‐NAb, and structural analysis post‐freeze‐drying revealed that both GelMA and GelMA‐GCA‐NAb exhibited a porous, irregular pore appearance(**Figure** **7**A). Degradation tests in PBS indicated similar degradation rates for both hydrogels over time(Figure 7B). Mechanical evaluation showed a slightly lower compressive modulus for GelMA‐GCA‐NAb compared to GelMA(Figure 7C). We assessed protein release in the leachate of GelMA‐GCA‐NAb hydrogel using a bicinchoninic acid (BCA) assay(Figure 7D). GelMA and GelMA‐GCA‐NAb exhibited non‐cytotoxicity and did not impact endothelial cell proliferation(Figure S8C, Supporting Information). Furthermore, GelMA‐GCA‐NAb retained its efficacy in ameliorating BMDC‐induced endothelial cell dysfunction (Figure S8A,B, Supporting Information).

Subsequently, we explored the efficacy of local GCA‐NAb administration on wound healing in diabetic mice (Figure 7E). GCA‐NAbs were coated with hydrogel and administered on excisional wounds to avoid proteolytic degradation and to control the release of GCA‐NAb in the wound areas.^[^ ^28^ ^]^ Topical GCA‐NAb administration significantly accelerated wound closure in diabetic mice (Figure 7F,G). Histological comparison of the wounds further confirmed enhanced wound closure in local GCA‐NAb‐treated wounds (Figure S9A, Supporting Information). Trichrome staining of the wound tissue revealed increased collagen deposits in the granulation tissue of local GCA‐NAb‐treated wounds (Figure S9B, Supporting Information). Consistently, the intensity of Ki67‐positive cells (Figure S9C,D, Supporting Information) and CD31‐positive cells (Figure 7H,I) increased in the GCA‐NAb‐treated wounds, indicating that topical administration of GCA‐NAb stimulates angiogenesis in diabetic mice. The mRNA levels of angiogenesis‐related genes significantly increased in the skin tissues of GCA‐NAb‐treated mice (Figure S9F). Similarly, skin perfusion significantly increased at the wound sites in GCA‐NAb‐treated mice (Figure 7J). Furthermore, topical treatment with GCA‐NAbs improved the inflammatory response at the wound site (Figure S9G, Supporting Information). Collectively, these data suggested that topical treatment with GCA‐NAb improved wound healing by promoting collagen deposition, epithelial regrowth, and angiogenesis.

### 2.10 GelMA‐GCA‐NAb Exhibits Superior Therapeutic Efficacy Compared to GCA‐NAb

We conducted a comparative analysis between localized GelMA‐GCA‐NAb and caudal vein infusion of GCA‐NAb, revealing the superior efficacy of GelMA‐GCA‐NAb in treating diabetic wounds (Figure S10A,B, Supporting Information). As the impact of GCA‐NAb on other organs remains unclear, the localized application of GelMA‐GCA‐NAb presents a reduced risk of systemic side effects. These findings offer valuable guidance for the subsequent clinical translation of GCA‐NAb in treating diabetic wounds.

## 3 Discussion

This study presents a novel report of myeloid cell‐derived GCA accumulation at wound sites and the potential contribution of GCA toward delayed wound healing in diabetes. GCA binds to the TRPM8 receptor to block downstream pathways in endothelial cells and thereby represses angiogenesis. Moreover, systemic or topical GCA‐NAb administration accelerates wound repair in diabetic mice.

GCA, which is highly expressed in myeloid cells, is an EF‐hand Ca2+ binding protein.^[^ ^29^ ^]^ EF‐hand proteins mediate various biological processes, including cell migration, apoptosis, and immune cell mobilization.^[^ ^30^ ^]^ However, the GCA‐deficient mice reproduced normally and seemed healthy, without any obvious abnormalities. GCA deficiency did not affect the generation of mature neutrophils in the bone marrow or immune recruitment.^[^ ^31^ ^]^ Thus, GCA is a potential therapeutic target for age‐related diseases, metabolic disorders, and tissue regeneration.

Angiogenesis is the primary step in tissue regeneration. Abnormal angiogenesis contributes to diabetes‐related wounds.^[^ ^32^ ^]^ In the present study, we found that GCA expression was significantly higher in animals and patients with diabetes than in the control groups. Both genetic and pharmacological inhibition of GCA improved angiogenesis and wound closure in diabetes. These results suggest an unequivocal role of GCA in angiogenesis and wound healing in diabetes. Endothelial functional integrity is indispensable for angiogenesis,^[^ ^33^ ^]^ which is typically impaired in diabetes.^[^ ^34^ ^]^ GCA deletion significantly improved the diabetes‐induced endothelial dysfunction. Additionally, GCA‐NAb markedly reversed the endothelial functional impairment, including migration and tube formation, in diabetes. Therefore, our findings establish that both genetic and pharmacological inhibition of GCA improves angiogenesis and accelerates wound healing by preserving endothelial function in diabetes.

We explored the molecular mechanisms underlying the function of GCA in diabetic wounds and identified TRPM8 as a functional receptor for endothelial cells. GCA bound to TRPM8, inactivated the ERK/HIF‐1/VEGF pathway, and impaired angiogenesis in diabetes. BMDC have been shown to effectively treat diabetes‐related vascular disease.^[^ ^35^, ^36^ ^]^ Diabetes is often accompanied by chronic inflammation and higher levels of myeloid cell‐derived proinflammatory secretomes are observed in diabetes and likely impair the therapeutic function of BMDCs.^[^ ^37^, ^38^, ^39^ ^]^ The increasing preclinical and clinical evidence have shown that BMDCs from diabetic patients lack sufficient therapeutic effects against diabetic vascular complications and even worsen the disease.^[^ ^40^, ^41^ ^]^ The proinflammatory secretome of BMDCs may contribute to vascular damage in diabetes whereas inhibition of myeloid cell‐derived inflammasomes improves angiogenesis and wound healing in diabetes.^[^ ^42^, ^43^ ^]^ Preconditioning strategies have been used to block the harmful secretome of BMDCs in diabetes, and this has successfully enhanced their performance in treating diseases.^[^ ^44^, ^45^ ^]^ Herein, we found that GCA inhibition significantly decreased the mRNA levels of inflammatory genes in the wound tissue. We have abundant reasons to believe that bone marrow cell‐derived GCA may function as a proinflammatory secretome in diabetes. GCA induces wound healing by impairing endothelial function and inhibiting angiogenesis. Thus, GCA may be a potential preconditioning target for restoring the functional characteristics of BMDCs in diabetes and constitutes an interesting research topic. This study had some limitations. First, the HFD/STZ models used in this study cannot fully mimic human diabetes. Second, although this study indicated an important direct contribution of the TRPM8 receptor, the data do not exclude other receptors that may mediate the effects of GCA on angiogenesis or wound healing. Finally, many other skin cells, such as fibroblasts, myofibroblasts, and keratinocytes, are involved in the wound healing process.^[^ ^46^ ^]^ However, the effects of GCA on other skin cells remain unknown.

In conclusion, our data show that GCA‐NAb could ameliorate the negative effects of GCA by promoting angiogenesis and wound healing in diabetes. Thus, GCA‐NAb‐based cleavage of GCA may be a novel therapeutic approach for wound closure.

## 4 Experimental Section

### Human Skin

The study recruited 16 patients (age 54 ± 9 years) with diabetic foot ulcers and 15 age‐matched normoglycemic control subjects (age 58 ± 7 years). All participants provided informed consent (Supplementary Information Table). This study was approved by the Medical Ethics Committee of Xiangya Hospital, Central South University.

### Animals

The 6–8 week‐old male C57BL/6J mice from Beijing Vital River Laboratory Animal Technology was obtained. The mice were housed in a specific pathogen‐free facility at the Laboratory Animal Research Center of Central South University, with a controlled temperature of 22–24°C and a 12 h light/dark cycle (lights on from 07:00 to 19:00). The animals were provided standard food and water ad libitum from the Hunan SJA Laboratory Animal Company (People's Republic of China), and their living environments were enriched to promote their well‐being.

Gca‐Lyz‐CKO and Gca‐KO mice were bred as previously described.^[^ ^17^ ^]^ GCA‐floxed and GCA‐knockout mice were generated using CRISPR/Cas9 technology at Bioray Laboratories (China). Lyz2‐Cre mice (Stock No004781‐B6.129P2‐Lyz2tm1(cre)Ifo/J) were obtained from Jackson Laboratory (USA). To generate myeloid‐specific GCa‐conditional KO mice, Gca^flox/flox^ mice were crossed with Lyz‐Cre mice. The resulting Lyz‐Cre;Gca^flox/+^ male offspring were backcrossed with unrelated Gca^flox/flox^ female mice. F2 Lyz‐Cre;Gca^flox/flox^(LyzCre;Gca^fl/fl^) mice exhibited an accelerated wound healing phenotype, whereas not all other offspring demonstrated this phenotype. Cre‐negative Gca^flox/flox^ mice served as controls. Genotyping was performed on all animals using PCR.

### Mice Dorsal Skin Wound Model

To induce wounds, mice were anesthetized with 1% pentobarbital sodium (5 µl g^−1^), and their dorsal hair was removed with an electric clipper and depilatory cream. Then, the skin was cleaned with PBS and two full‐thickness wounds were created on the dorsal region of the mice using a 6‐mm biopsy punch.^[^ ^47^ ^]^ Digital photographs with a ruler included for distance correction were taken on the day of surgery and every other day thereafter. The wound area was measured using ImageJ.

### Animal Experimental Procedures

To establish the type 2 diabetes mellitus model, 8‐week‐old mice were fed a high‐fat diet for 4 weeks, followed by 5 consecutive days of intraperitoneal injections of streptozocin (STZ,50 mg k^−1^g).^[^ ^48^ ^]^ After an 8‐week period, to ensure the successful establishment of the diabetic model, diabetic wound mapping was initiated. Wild‐type diabetic mice were randomly divided into treatment and control groups, with the treatment group receiving GCA‐Nab (3 mg k^−1^g) injections via the tail vein every 3 days and the control group receiving PBS injections.

### Histochemistry, Immunohistochemistry, and Immunofluorescence Staining Analysis

Histological and immunohistochemical analyses were performed as previously described.^[^ ^49^, ^50^, ^51^ ^]^ The skin samples were fixed in 10% paraformaldehyde for 24 h and subsequently embedded in paraffin and sectioned to a thickness of 5 µm. For histological examination, paraffin sections were deparaffinized, rehydrated, and stained with hematoxylin and eosin and Masson's trichrome stain. Furthermore, antigen retrieval was performed on skin tissue sections for immunohistochemical and immunofluorescence staining. The following reagents were used: hematoxylin–eosin (HE) Stain Kit (Solarbio), Masson's Trichrome Stain Kit (Solarbio), Sodium Citrate Antigen Retrieval Solution (Solarbio), anti‐ki67 (1:1000, Proteintech),anti‐cd31 (1:500, Proteintech), DAPI (Solarbio), and anti‐GCA (1:200, Invitrogen)F(ab′)2‐Goat anti‐Rabbit IgG (H+L) Cross‐Adsorbed Secondary Antibody, Alexa Fluor 555(1:500, Invitrogen), F(ab′)2‐Rabbit anti‐Goat IgG (H+L) Cross‐Adsorbed Secondary Antibody, Alexa Fluor 488(1:500, Invitrogen).

### Cell Culture

After euthanizing the mice, bone marrow was removed by washing the cells with DMEM. Red blood cells were lysed and the remaining cells were washed with DMEM supplemented with 10% FBS. Isolated BMDCs were washed with DMEM complete medium and cultured in a cell culture incubator.^[^ ^52^ ^]^

The Human Umbilical Vein Endothelial Cells (HUVEC) were purchased from the American Type Culture Collection (Rockville, MD, USA) and maintained in ECM (ScienCell, USA) containing 5% FBS, 1% endothelial cell growth supplement (ECGS), and 1% P/S. Cells were incubated in a humidified atmosphere containing 5% CO_2_ at 37°C.

### Plasmid Transfection and RNA Interference

Plasmids encoding myc‐tagged GCA and FLAG‐tagged TRPM8 were procured from Tsingke (Beijing, China) and transfected into cells using Lipofectamine 2000 reagent (Invitrogen) as per the manufacturer's instructions. For the knockdown experiment, cells were seeded in 6‐well plates and transiently transfected with 30 pmol siRNA oligonucleotides using Lipofectamine RNAiMAX Transfection Reagent (Invitrogen) according to the manufacturer's instructions. The Si‐Trpm8 and the corresponding negative control siRNAs were obtained from RIBOBIO (Guangzhou, China).^[^ ^53^ ^]^

### Establishment of the HUVEC–BMC Co‐Culture System

After inoculating BMDCs isolated from WT or KO diabetic mice in the lower chamber of the Transwell chambers, the BMDCs were co‐cultured with HUVECs inoculated in the upper chamber for 24 h (the upper and lower chambers contained 1% and 5% fetal bovine serum (FBS), respectively). The number of migrating HUVECs was subsequently determined using crystal violet staining.^[^ ^54^ ^]^

### In Vitro Angiogenesis Assay

HUVECs were seeded at a density of 5 × 10^4^ cells per well in a 48‐well plate precoated with 150 µL of EC‐Matrigel per well (catalog no. ECM625; Chemicon). Tube formation was quantified 6 h after various treatments or siRNA transfections by analyzing the sprouting tube‐like structures in five randomly selected fields at 40 × magnification using an inverted phase‐contrast microscope.

### Scratch and Migration Experiments

HUVECs were seeded in 6‐well plates that were pre‐coated with collagen overnight. After the cells were fully spread out, markers were made by scratching the surface of the well with a 200 µL micropipette tip. The wells were washed once with PBS and filled with ECM containing 0.5% FBS and 1% PS for culturing. Images were captured at 0 and 12 h to record the size of the scratches, which were then analyzed quantitatively using ImageJ.^[^ ^55^ ^]^ Migration assays were conducted using Transwell chambers with 8 µm polycarbonate filters (BD Biosciences, San Jose, CA) and a medium containing 5% FBS in the lower chambers as a chemoattractant. Next, 5 × 10^4^ cells were suspended in 500 µL serum‐free medium and placed in the upper chamber, which was then incubated at 37°C for 24 h. Following incubation, cells that migrated through the filter were counted in six randomly selected fields using crystalline violet staining. The mean number of cells per field was recorded. Each assay was performed on duplicate filters and each experiment was repeated three times to ensure accuracy.^[^ ^56^ ^]^

### GCA and GCA‐NAb Assay

BMDCs were isolated from wild‐type and CKO mice, seeded at a density of 1×10^6^ cells in 12‐well plates, and cultured for 24 h. The supernatants were collected after centrifugation and used for subsequent analyses. For the HUVEC tube‐formation assay, 10% of the collected supernatant was added to the culture medium along with GCA‐NAb (100 ng mL^−1^). The effect of GCA‐NAb was evaluated by monitoring the number of tubes formed. In the cell experiments related to GCA, a concentration of 100 µM GCA was used.

### Immunoprecipitation and Western Blot Analysis

Immunoprecipitation was performed as described previously.^[^ ^57^ ^]^ Briefly, total cell lysates were obtained and subjected to immunoprecipitation using antibodies specific for FLAG (14 793, 1:200; Cell Signaling Technology) and MYC (2276S, 1:200; Cell Signaling Technology), followed by adsorption to protein G Sepharose. The resulting immunoprecipitates were resolved using SDS‐PAGE and transferred onto polyvinylidene difluoride (PVDF) membranes (Bio‐Rad Laboratories). The membrane was then probed with antibodies against MYC (2276S, 1:2000, Cell Signaling Technology) and FLAG (14 793, 1:2000, Cell Signaling Technology), and visualized using enhanced chemiluminescence (ECL Kit; Amersham Biosciences).

For Western blot analysis, total cell lysates were separated using SDS‐PAGE and transferred onto PVDF membranes (Bio‐Rad Laboratories).^[^ ^58^ ^]^ The membranes were probed with specific antibodies against GCA (PA5‐77127, 1:1000, Invitrogen), TRPM8 (DF7966, 1:2000, Affinity), ERK (4695T, 1:2000, Cell Signaling Technology), p‐ERK (4370S, 1:2000, Cell Signaling Technology), HIF‐1α (3281T, 1:2000, Cell Signaling Technology), and VEGF (50661S, 1:2000, Cell Signaling Technology), and GAPDH (5174, 1:2000, Cell Signaling Technology), and subsequently reprobed with the appropriate horseradish peroxidase‐conjugated secondary antibodies. The blots were visualized using an ECL kit (Amersham Biosciences).

### qRT‐PCR Analysis

Quantitative real‐time PCR (qRT‐PCR) was performed according to a previously established protocol.^[^ ^59^ ^]^ Briefly, total RNA was extracted from skin tissues or cultured cells using the TRIzol reagent (Invitrogen) according to the manufacturer's instructions. RNA quality was assessed by analyzing the 260:280 ratio using a NanoDrop2000 spectrophotometer (Thermo Scientific, Waltham, MA, USA). Samples with a ratio of 1.8–2.0 were further processed for analysis. cDNA was synthesized by reverse transcription of the total RNA using SuperScript II (Invitrogen). Quantitative real‐time PCR was performed on an Applied Biosystems 7300 system (Life Technologies, Warrington, U.K.) using the SYBR Green PCR Master Mix (PE Applied Biosystems). GAPDH was used as an internal control, and gene expression levels were calculated using the DCt method.

### Single‐Cell RNA‐Seq Data Quality Control, Dimension‐Reduction, and Clustering

Before analysis, the implemented a filtering step in which cells with UMI counts below 30000 and gene counts ranging from 200 to 5000 were excluded. Cells with > 50% mitochondrial content were excluded. Following filtration, we utilized the Seurat v3.1.2 functions for dimension reduction and clustering. Gene expression was normalized and scaled using the NormalizeData and ScaleData functions. The top 2000 variable genes were selected for PCA using the FindVariableFeatures function. Using the foremost 20 principal components, the cells were segregated into distinct clusters using FindClusters. We addressed the batch effects among the samples using harmony. Finally, the UMAP algorithm was employed to visualize the cells in a two‐dimensional space.^[^ ^60^, ^61^ ^]^

### Bioinformatics Analysis

Single‐cell sequencing data (Figure S1, Supporting Information) was obtained from the Tabula Muris library and subsequently analyzed using an online platform (https://tabula‐muris.ds.czbiohub.org/).

### Preparation of GelMA‐GCA‐NAb Hydrogels

The synthesis of GelMA‐GCA‐NAb hydrogels was conducted according to the specifications of Gelatin Methacryloyl (GelMA, Engineering For Life, China) and AC‐PEG‐NHS(Engineering For Life, China). To prepare the hydrogel, the LAP photoinitiator was added to a GCA‐NAb solution along with AC‐PEG‐NHS. The mixture was then incubated in a light‐proof environment at the appropriate temperature for 24 h before being combined with the GelMA solution.GCA‐NAb(0.2 mg ml^−1^) was obtained after mixing. The resulting mixture was subjected to ultraviolet (UV) light to form a hydrogel.^[^ ^62^ ^]^

### Hydrogel Performance Testing

To assess the release rate of GCA‐NAb from GelMA‐GCA‐NAb, the hydrogel was prepared and immersed in PBS at 37°C. Supernatant samples were collected on days 0, 2, 4, 6, and 8, and their concentrations were measured using the BCA method. Simultaneously, the prepared hydrogels were immersed in PBS at 37°C, and their weights were measured every 3 days to generate the dissolution curve for the hydrogel. The mechanical properties of GelMA and GelMA‐GCA‐NAb scaffolds were assessed using disks measuring 13 mm in diameter and 55 mm in thickness before testing. Tensile tests were conducted using a mechanical testing apparatus(CMT6103 MTS Meters Industrial Systems). The structures of GelMA and GelMA‐GCA‐NAb hydrogels were examined using a scanning electron microscope (TESCAN MIRA LMS).

### Statistical Analysis

Data is presented as mean ± SD. Data showed a continuous normal distribution. Two‐tailed Student's *t* test was used for comparisons between two groups, and one‐way ANOVA were used for comparisons among multiple groups (ns, not significant; ^*^ *p* < 0.05; ^**^ *p* < 0.01; ^***^ *p* < 0.001; ^****^ *p* < 0.0001).

## Conflict of Interest

The authors declare no conflict of interest.

## Author Contributions

P.X. and M.J. contributed equally to this work. Z.H. and Z.Y. designed the projects and supervised this study. J.M. and X.P. performed most of the experiments and analyzed the data. X.P. participated in the experimental work and data analysis. J.M. and X.P. drafted the manuscript. L.X. and Z.Y. edited the manuscript. All authors have read and approved the article.

## Supporting information

Supporting Information

## Data Availability

The data that support the findings of this study are available in the supplementary material of this article.
