# Supplementary material for: Targeting Grancalcin Accelerates Wound Healing by Improving Angiogenesis in Diabetes
Source: Adv Sci (Weinh). 2024 Feb 2;11(14):2305856. doi: 10.1002/advs.202305856 (PMC11005700; doi:10.1002/advs.202305856)
Supplement: Supplementary file 1 — Supporting Information [file ADVS-11-2305856-s001.pdf]

ADVANCED SCIENCE
Open Access

Supporting Information

for *Adv. Sci.*, DOI 10.1002/advs.202305856

Targeting Grancalcin Accelerates Wound Healing by Improving Angiogenesis in Diabetes

Peng Xiang, Meng Jiang, Xin Chen, Linyun Chen, Yalun Cheng, Xianghang Luo, Haiyan Zhou and Yongjun Zheng**

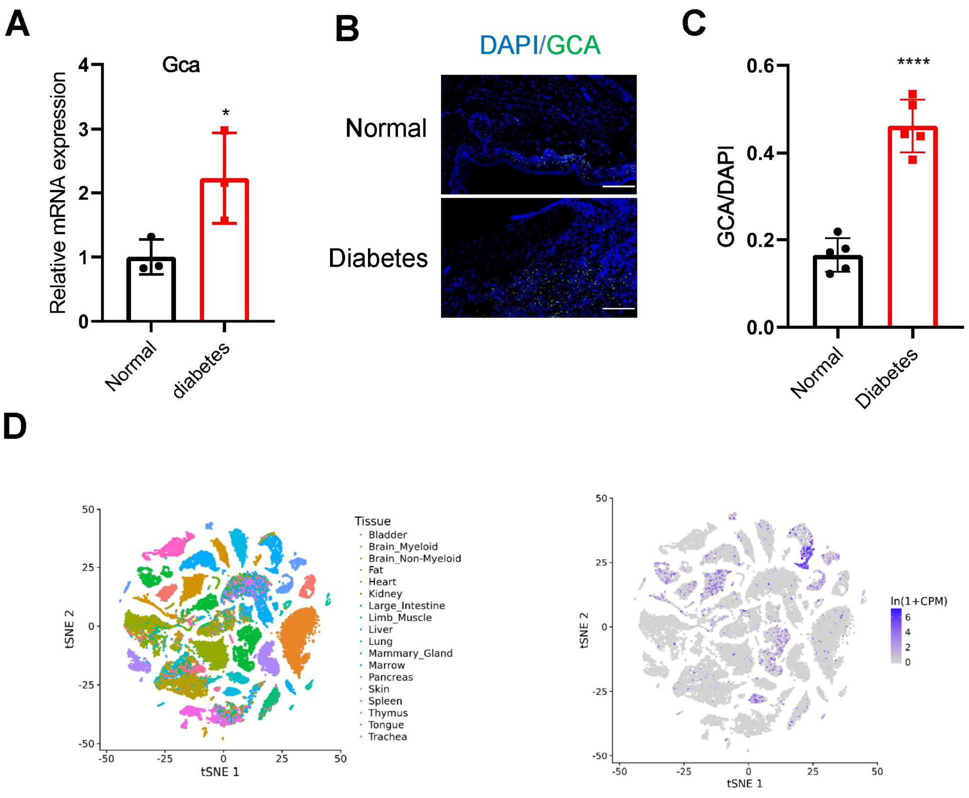

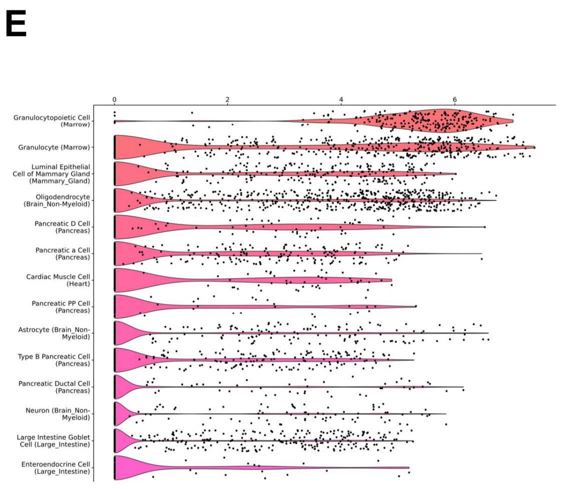

Tissue	Mean	Median	Median > 0	# cells	% 0	% > 0
Granulocytopoietic Cell (Marrow)	5.30	5.53	5.54	221	1.81	98.19
Granulocyte (Marrow)	2.26	0.00	5.06	761	51.51	48.49
Luminal Epithelial Cell of Mammary Gland (Mammary_Gland)	1.36	0.00	3.86	578	63.15	36.85
Oligodendrocyte (Brain_Non-Myeloid)	1.25	0.00	4.40	1574	68.61	31.39
Pancreatic D Cell (Pancreas)	1.22	0.00	3.22	140	60.71	39.29
Pancreatic a Cell (Pancreas)	1.19	0.00	2.94	390	58.46	41.54
Cardiac Muscle Cell (Heart)	1.11	0.00	3.35	133	66.17	33.83
Pancreatic PP Cell (Pancreas)	0.96	0.00	2.60	73	64.38	35.62
Astrocyte (Brain_Non-Myeloid)	0.96	0.00	3.34	432	71.99	28.01
Type B Pancreatic Cell (Pancreas)	0.95	0.00	2.80	449	66.15	33.85
Pancreatic Ductal Cell (Pancreas)	0.94	0.00	3.36	161	71.43	28.57

Figure S1. GCA enriched in myeloid cells

(A) mRNA expression of GCA in skin tissues of normal and diabetic mice (n=3). Immunofluorescence staining (B) and quantitative analysis (C) of GCA in mouse skin tissue (n=5; scale bars, 200 μm). (D) Bioinformatic analysis of scRNA-seq of GCA from mice. (E) Violin plots and list of GCA-related gene expression in mouse cell populations. Data were shown as the mean ± SD. *p <0.05, ****p < 0.0001.

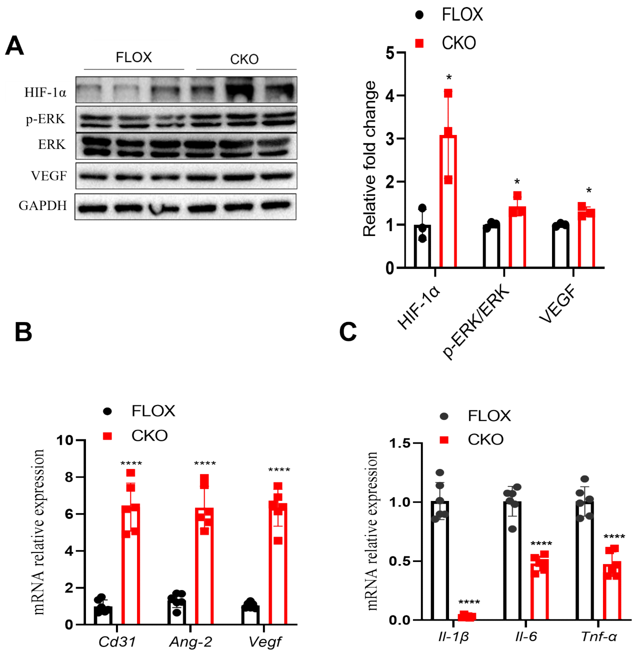

Figure S2. Myeloid-specific knockout of GCA improves vascularization and inflammation in murine diabetic wounds

(A)Representative Western blot images depicting HIF-1, ERK, p-ERK, and VEGF levels were obtained from the wounds of Flox and CKO diabetic mice at 7 days post-wounding, along with the quantification of the results(n=3). mRNA expression of Cd31, Ang-2, Vegf (B) and inflammation-related genes (C) in wound tissues of Flox and CKO mice(n=6). *p < 0.05, ****p < 0.0001.

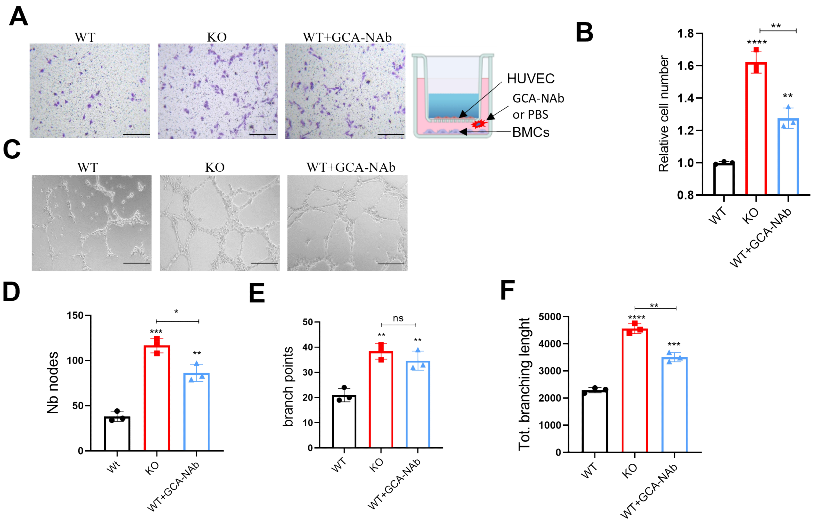

Figure S3. GCA-neutralizing antibody ameliorates angiogenic activity

(A) Transwell migration assays were performed by counting the migrated HUVEC co-cultured with BMDCs isolated from WT or KO diabetic mice and treated with PBS or GCA-NAb.(B) Quantitative analysis of HUVEC migration(n=3,scale bars, 100 μm); (C–F) HUVECs were cultured with BMDCs supernatant from WT or KO diabetic mice co-cultured with PBS or GCA-NAb(n=3). Tube formation (C) was examined after 6 h(scale bars, 100 μm); the Nb nodes (D), total number of branch points (E) and total branching length(F) were measured and analyzed using ImageJ (n=3). Data were shown as the mean ± SD. *p < 0.05, **p < 0.01, ***p < 0.001.

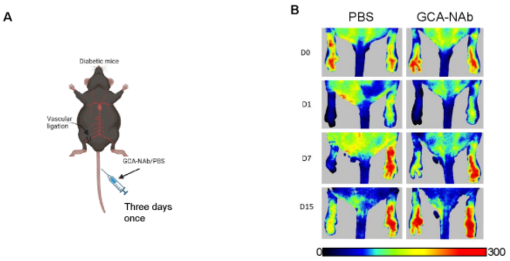

Figure S4. GCA-NAb promotes blood-flow recovery in diabetic mice

Restoration of blood flow was evaluated after surgically inducing hindlimb ischemia in

diabetic mice. (A) Mouse hindlimb ischemia model. (B) Representative laser Doppler images on days 0, 1, 7, and 15.

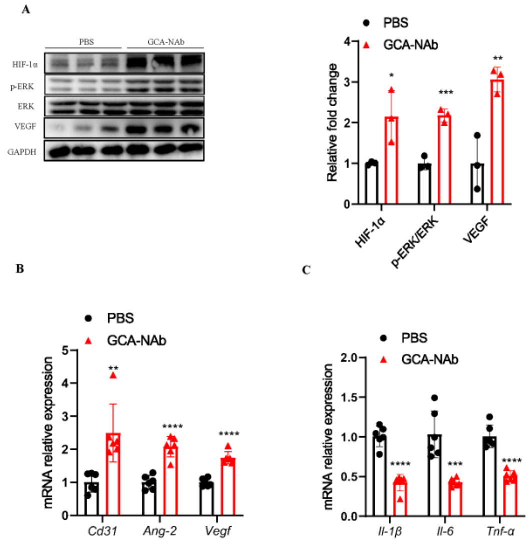

Figure S5. GCA-NAb improves vascularization and inflammation in wounds of diabetic mice

(A) Representative Western blot images depicting HIF-1, ERK, p-ERK, and VEGF levels were obtained from wounds of diabetic mice and the quantification of the results(n=3). mRNA expression of Cd31, Ang-2, Vegf (B) and inflammation-related genes (C) in the wound tissue of diabetic mice(n=6). Data were shown as mean ± SD. *p < 0.05, **p < 0.01, ***p < 0.001, ****p < 0.0001.

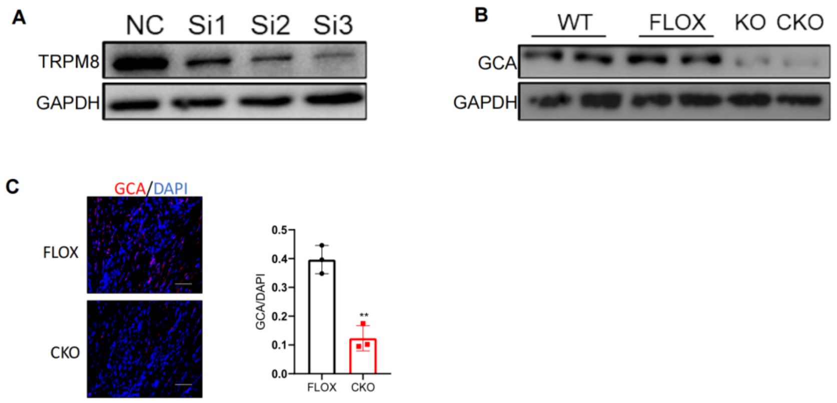

Figure S6. Validation of genetic mice and small-interfering RNA

(A) Representative Western blot analysis of TRPM8 after siRNA-Trpm8 treatment. (B) GCA expression in the mouse bone marrow of gene mice. (C) Immunofluorescence staining of GCA in wounds (n=3; scale bars, 50 μm).

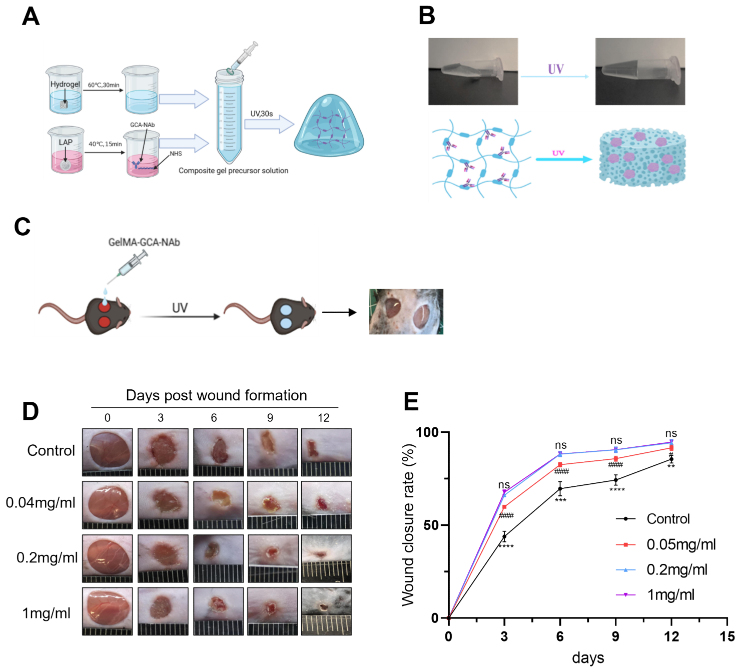

Figure S7. Flowchart of GelMA-GCA-NAb hydrogel construction

(A) Schematic representation of the methodology used for the preparation and manufacturing of hydrogels. (B) Photograph (top) and Model Diagram (bottom) of GelMA-GCA-NAb solution and GelMA-GCA-NAb hydrogel formation after UV light irradiation. (C) Method chart of GelMA-GCA-NAb hydrogel for wound healing. (D-E) Representative images of wounds with different concentrations of GelMA-GCA-NAb hydrogel (D) and wound-healing rates (E) at days 0, 3, 6, 9,and 12. ns,not significant. 0.2mg/ml vs 1.0mg/ml; $^{**}p < 0.01$, $^{***}p < 0.001$, $^{****}p < 0.0001$, 0.05mg/ml vs control; $^{\#}p$ <0.001, $^{\#\#\#\#}p < 0.0001$, 0.2mg/ml vs 0.05mg/ml.

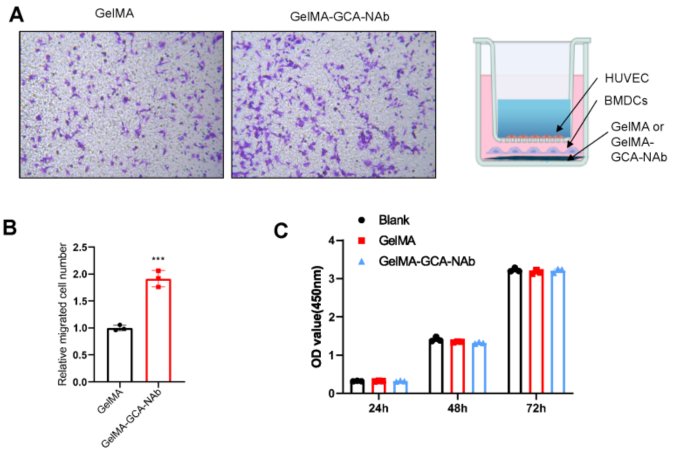

Figure S8. GelMA has no impact on the biological function of GCA-NAb

(A) Transwell co-culture model: BMDCs and hydrogel were seeded into the lower chamberand and HUVECs were placed in the upper chamber (right). Transwell migration assays were performed by counting the migrated HUVEC co-cultured with BMDCs isolated from diabetic mice and treated with GelMA or GelMA-GCA-NAb(left). (B) Quantitative analysis of HUVEC migration(n=3,scale bars, 100 μm); (C) The proliferation of HUVECs cultured directly on various hydrogels was assessed using the CCK-8 kit(n=3).Data were shown as mean ± SD. ***p < 0.001.

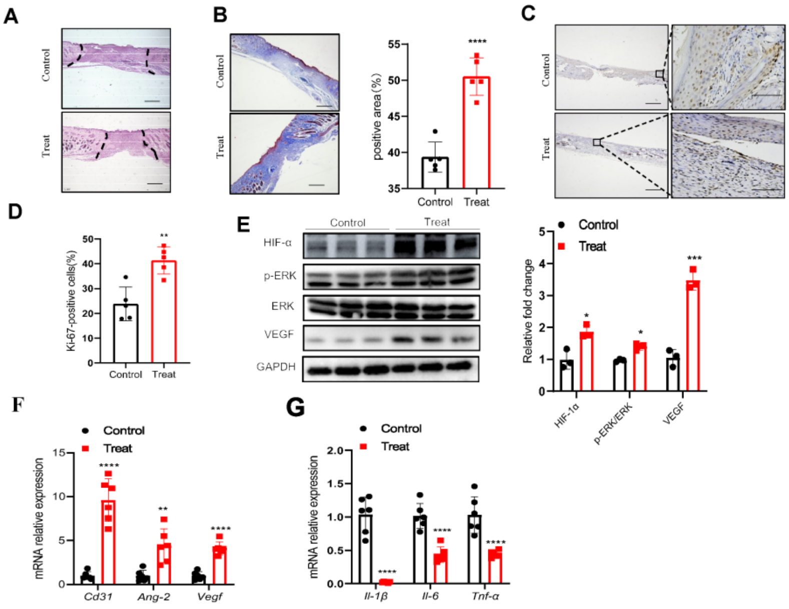

Figure S9. GelMA-GCA-NAb hydrogel enhances vascularization and mitigates

inflammation in wounds of diabetic mice. (A) Representative hematoxylin–eosin-stained wound sections on day 12 (scale bars, 200 μm). (B) Representative image of Masson's trichrome staining of the wound sections on day 12 (scale bars, 200 μm). (C-D) Immunohistochemical staining (C) and quantitative analysis (D) of Ki67 expression in the wound sections on day 12 (scale bars, 200 and 50 μm). (E) Representative Western blot images for HIF-1, ERK, p-ERK, and VEGF levels were obtained from wounds of diabetic mice and the quantification of the results. (F-G) mRNA expression of Cd31, Ang-2, Vegf (F) and inflammation-related genes (G) in the wound tissue of diabetic mice. Data were shown as mean ± SD. *p < 0.05,**p < 0.01, ***p < 0.001, ****p < 0.0001.

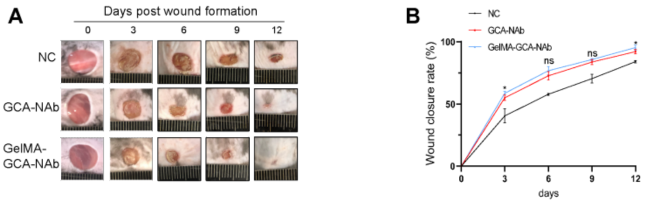

Figure S10. GelMA-GCA-NAb exhibits superior therapeutic efficacy compared to GCA-NAb

(A-B) Representative images (A) of diabetic wounds treated with GCA-NAb or GelMA-GCA-NAb and quantitative analysis of wound healing rates (B) at days 0, 3, 6, 9, and 12. Data were shown as mean ± SD. ns, not significant, *p < 0.05, GCA-NAb vs GelMA-GCA-NAb.

KEY RESOURCES TABLE

Antibodies	*Source*	*Identifier*
Anti-GCA	Invitrogen	Cat# PA5-77127
Anti-GAPDH	Cell Signaling Technology	Cat# 5174
Anti-MYC	Cell Signaling Technology	Cat# 2276
Anti-ERK1/2	Cell Signaling Technology	Cat# 4695T
Anti-p-ERK1/2	Cell Signaling Technology	Cat# 4370S
Anti-HIF-1α	Cell Signaling Technology	Cat#3281T
Anti-VEGF	Cell Signaling Technology	Cat#50661S
Anti-FLAG	Cell Signaling Technology	Cat#14793
Anti-β-Actin	Cell Signaling Technology	Cat#3700s
Anti-KI67	Proteintech	Cat#28074-1-AP
Anti-CD31	Proteintech	Cat# 28083-1-AP
Donkeyanti- Rabbit Alexa fluor 488	invitrogen	Cat#A32794
Donkey anti- Rabbit Alexa fluor 555	invitrogen	Cat#A32790

Critical commercial assays		
IHC Kit	ZSGB-BIO	Cat# pv-6002
Masson's Trichrome Stain Kit	Solarbio	Cat# G1340
Hematoxylin-Eosin (HE) Stain Kit	Solarbio	Cat# G1120
DAPI solution	Solarbio	Cat# C0065
Gelatin Methacryloyl (GelMA)	Engineering For Life	Cat# EFL-GM-60
Acrylate-PEG-NHS(AC-PEG-NHS)	Engineering For Life	Cat# EFL-AC-PEG-NHS-2K

Experimental models: Cell and		
HEK293T	ATCC	CRL-3216
HUVECs	ATCC	PCS-100-010
C57/BL6J mice	Vital River Laboratory Animal Technology	N/A
GCA-knockout mice	This paper	N/A
Lyz2-Cre mice	Jackson Laboratory	004781-B6.129P2-Lyz2$^{tm1(cre)Ifo}$/J

Recombinant DNA		
Myc-GCA plasmid	This paper	N/A
FLAG-TRPM8 plasmids	This paper	N/A

Deposited data		
Mass spectrometry proteomics data	This paper	N/A
scRNA-seq	This paper	https://tabula-muris.ds.czbiohub.org
scRNA-seq	This paper	N/A

Deposited data		
ImageJ	NIH	https://imagej.nih.gov/ij/
PIMsoft	Valsoft	https://www.pimsoft-group.com/

Primers used for SYBR green quantitative real-time PCR

Human-CD31-F	AACAGTGTTGACATGAAGAGCC
Human-CD31-R	TGTAAAACAGCACGTCATCCTT
Human-Gapdh-F	GGAGTCCACTGGCGTCTTCAC
Human-Gapdh-R	GAGGCATTGCTGATGATCTTGAGG
Human-Gca-F	TGCATTCAAAGAGCTATGGGC
Human-Gca-R	CTGTGCCACTTCCATCTTGAT
Human-Icam1-F	TTGGGCATAGAGACCCCGTT
Human-Icam1-R	GCACATTGCTCAGTTCATACACC
Human-Trpm8-F	CAGAAGGAATGACACTCTGGAC
Human-Trpm8-R	TCACCAAGTCGCTTTCACTGT
Human-Vacm1-F	TTTTATGCGCCCGATTGTTTTG
Human-Vacm1-R	TTGCTGGGCCTTTATCATCCC
Human-Vegf-F	AGGGCAGAATCATCACGAAGT
Human-Vegf-R	AGGGTCTCGATTGGATGGCA
Lyz-cre-F1	CTTGGGCTGCCAGAATTTCTC
Lyz-cre-R1	CCCAGAAATGCCAGATTACG
Mouse-Ang2-F	AGAATAAGCAAGTCTCGCTTCC
Mouse-Ang2-R	TGAACCCTTTAGAGGCTCGGT
Mouse-Cd31-F	ACGCTGGTGCTCTATGCAAG
Mouse-Cd31-R	TCAGTTGCTGCCCATTCATCA
Mouse-Gapdh-F	TCAACAGCAACTCCCACTCTTCCA
Mouse-Gapdh-R	TTGTCATTGAGAGCAATGCCAGCC
Mouse-Gca-F	GGGGCGTTTGGAAACTTCAG
Mouse-Gca-R	AGGGGAGTAGCTGTCAGAATAAC
Mouse-Il1β-F	CCGTGGACCTTCCAGGATGA
Mouse-Il1β-R	GGGAACGTCACACACCAGCA
Mouse-Il6-F	ACTCCAGAAGACCAGAGGAAAT
Mouse-Il6-R	CCAGAGATACAAAGAAATGATGG
Mouse-Tnfα-F	AGCCCCCAGTCTGTATCCTT
Mouse-Tnfα-R	CTCCCTTTGCAGAACTCAGG
Mouse-Trpm8-F	CCAAGGAGTTTCCAACAGACG
Mouse-Trpm8-R	CGTGGCTTCAAAGCAAAGTTT
